# Maternal, paternal, and peer attachment and adolescent behavior problems: a validation study of the Inventory of Parent and Peer Attachment (IPPA-45)

**DOI:** 10.1186/s40359-025-03643-7

**Published:** 2025-12-03

**Authors:** Mojtaba Habibi Asgarabad, Pardis Salehi Yegaei, Nazanin Seyed Yaghoubi Pour, Ebrahim Khodaie, Ross B. Wilkinson, Nora Wiium

**Affiliations:** 1 Norwegian University of Science and Technology, Trondheim, Norway; 2 Department of Psychology, Faculty of Humanities and Social Sciences, Istinye University, Istanbul, Turkey; 3 Iran University of Medical Science, Tehran, Islamic Republic of Iran; 4 University of Tabriz, Tabriz, Islamic Republic of Iran; 5 University of Tehran, Tehran, Islamic Republic of Iran; 6 University of Newcastle Australia, Newcastle, Australia; 7 University of Bergen, Bergen, Norway

**Keywords:** Adolescence, The inventory of parent and peer attachment, Internalizing and externalizing behavior problems, Academic performance, Gender invariance, Validity, Reliability

## Abstract

**Background:**

This study was conducted to assess the psychometric soundness of the short form of the Inventory of Parent and Peer Attachment (IPPA-45) and its relationship with internalizing and externalizing behavior problems.

**Methods:**

Iranian adolescent girls and boys aged 14–17 (*n* = 1532; 50% girls; Mean _age_ = 15.50, SD = .97) were asked to report their attachment security, internalizing and externalizing behavior problems (Youth Self-Report), and demographic characteristics.

**Results:**

Results of the confirmatory factor analysis supported the original three-factor first-order model for maternal, paternal, and peer forms, with equivalency across gender and reasonable reliability. Internal construct validity was supported by acceptable correlation coefficients among the three dimensions. The trust and communication subscales of the IPPA-45 were negatively correlated with all subscales of behavioral problems and adolescents’ age, while positively correlated with academic performance. Conversely, the alienation subscale showed significant but weaker correlations with these variables in the opposite direction. Satisfactory discriminant validity was demonstrated through Average Variance Extracted (AVE) for trust and communication, but not for alienation. Gender discrepancies were observed, with boys exhibiting higher attachment security to their parents and girls displaying stronger attachment to their peers.

**Conclusions:**

The findings suggest that the IPPA-45 is a reliable and valid instrument for assessing adolescents’ attachments to their parents and peers. Thus, attachment relationships, including peer attachment, appear to be essential for protecting teenagers from behavior problems and should be targeted in intervention programs.

## Introduction

Attachment theory describes the key motivations and processes that underlie close interpersonal relationships with significant others throughout childhood and into adulthood [3, 9]. The dominant attachment figure for children is their parents. However, attachment gradually shifts to peers, as teens begin to seek intimacy and support beyond their immediate family [30, 56]. Armsden and Greenberg [6] suggested that peer-to-peer attachments hold the same significance as parental relationships in adolescents’ psychological well-being. Their hypothesized model of attachment comprised three factors: trust, communication, and alienation. While alienation pertains to the negative emotional/cognitive encounters related to unresponsive or insecure attachment figures, trust and communication are described as positive emotional/cognitive encounters related to responsive and secure attachment figures [6].

The self-report Inventory of Parent and Peer Attachment (IPPA) was developed by Armsden and Greenberg [6] to estimate adolescents’ perceptions of relationship quality and security with their parents and peers. An abbreviated version (IPPA-45 [79]) includes 15 items each for mother, father, and peer attachment. A preliminary Confirmatory Factor Analysis (CFA) was conducted by Wilkinson and Goh [79] on English-speaking adolescents supported the three-factor model (trust, communication, and alienation). Although widely used in different contexts (e.g., the United States [74], Australia [16], and Spain [53]), the factor structure of IPPA-45 has not been re-evaluated since its development.

The IPPA-45 has demonstrated high internal consistency, with initial Cronbach’s alphas of 0.93 for both mother and father attachment, and 0.89 for peer attachment. Likewise, inter-correlation among the three dimensions appeared to be moderate to strong (*r* = 0.65–0.91 for fathers, 0.69–0.90 for mothers, and 0.36–0.86 for peers) [79]. Other studies have also reported adequate internal reliability [16, 53].

### Attachment and adjustment in adolescents

Research has confirmed that secure attachment to both peers and parents acts as a protective factor against internalizing and externalizing behavior problems [24, 48, 53, 75], issues in gender identity development, such as lower gender contentedness and higher pressure for gender differentiation [18], suicidal intention, and resentment severity [67].

The influence of attachment insecurity on behavior may be explained through different underlying mechanisms: 1) insecure attachment reduces self-esteem [5, 62] and positive self-image [46], which fosters anxiety/depression [57] or aggression [54] as means of expressing frustration,2 insecure attachments hinder social skill acquisition, such as conflict resolution [11], sympathy [65], and cooperation [19], and limit access to social support [48], resulting in anxiety [34, 35] or bullying perpetration [12],3 insecure attachments leave children feeling unsupported, leading to a sense of frustration [81] and difficulty managing emotions [65]. This increases the likelihood of anxiety/depression or aggression [44],4 secure attachments promote a positive dynamic process in which children feel supported and respond by behaving appropriately, where children feel supported and reciprocate by behaving in appropriate ways. Insecure attachments, in contrast, can create negative cycles of misbehavior and punitive responses, thereby exacerbating behavioral problems [75].

Existing evidence has documented gender-specific patterns in attachment to parents and peers, with boys reporting stronger attachment to parents and girls demonstrating greater attachment to peers [78]. On the IPPA-45 subscales, girls reported greater alienation toward their fathers, whereas boys scored marginally higher on trust [79]. However, drawing inferences from differences in group means before verifying measurement equivalence between groups may lead to misleading results [43, 72]. Thus, to ensure that difference in subscales means across gender reflects actual gender-specific patterns and not merely differences in how male and female respondents perceive them, it is required first to confirm the factorial invariance of the measure across genders. Although Wilkinson and Goh [79] found the final model to be invariant across gender, it should be established in other cultural contexts.

### Attachment across cultures

Cross-cultural studies (e.g., [71]) have revealed the generalizability of a nomological network of attachment-related constructs across cultures. Evidence from Iran, consistent with other countries, has shown links between attachment security and social skills (Ghorbanian, PourEbrahim, et al., 2016), social adjustment and academic performance [27, 28], smoking [36, 40], drug use tendency [37], and chronic pain [4]. Nevertheless, investigating the psychometric soundness of the IPPA-45 and its association with behavioral problems in Iranian adolescents is essential as previous evidence highlights the cultural significance of family relationships in Iran [34, 35], where parent-adolescent bonds play a critical role in emotional and psychological support within a collectivist framework [66]. As modernization and shifting family dynamics impact these relationships [52], understanding attachment security in Middle Eastern countries is crucial for addressing rising mental health challenges [34, 35], such as behavioral problems [25, 50]. Most research on the IPPA is based on data from Western samples of adolescents. While some studies have found cross-cultural support for the invariance of the IPPA across English and non-English-speaking samples (e.g., [73]) and Western and Asian samples [47]. There is some evidence that the original IPPA factor structure is not always replicable in non-English-speaking populations. For example, Zulkefly and Wilkinson [82] proposed an alternative three-factor structure for the parental scales and a two-factor structure for the peer scale in a large sample of Malaysian adolescents. It is thus necessary to address the lack of culturally adapted tools and research gaps on adolescent attachment in Middle Eastern societies, to guide interventions aimed at improving adolescents’ behavioral outcomes in Iran.

### The present study

Given the gaps and inconsistencies in the extant literature, the present study used a sample of Iranian adolescents to 1) test the construct validity of the three-dimensional structure of the IPPA-45 [79] and its internal construct validity,2) evaluate its internal consistency; 3) examine the convergent and discriminant validity of the IPPA-45 via its association with behavior problems (i.e., internalizing and externalizing problems), academic performance, and age; and 4) explore the gender differences in the level of each attachment aspect and their measurement invariance across gender. Based on earlier research, we hypothesized that behavior problems and age would be negatively correlated, and academic performance would be positively correlated with attachment, trust, and communication. In contrast, behavior problems and age would be positively associated, and academic performance would be negatively associated with attachment alienation.

## Materials and methods

### Participants

In the present cross-sectional study, 1,532 high school student volunteers (765 boys and 767 girls) aged between 14 and 17 years (Mean = 15.50, SD = 0.97) were recruited from six boys’ schools and three girls’ schools in Tehran, Iran. Based on CFA guidelines, a minimum of 10–20 participants per parameter is required to achieve power ≥ 0.80 at *α* = 0.05 [45]. The final sample far exceeded this threshold to ensure reliable model estimation and subgroup analyses. Eligibility criteria required students to be between 14 and 18 years old and currently enrolled in high school. Participants were selected using a convenience sampling method and were distributed across the ninth (15.5%), tenth (37.8%), eleventh (27.8%), and twelfth (19%) grade levels. Regarding parental education level, 3.6% of fathers did not hold an official degree, 74% had a diploma or lower level of education, and 22.4% held an academic degree. Similarly, 4.2% of mothers did not have official degrees, 76.8% had a diploma or lower level of education, and 19% held an academic degree. Approximately 95.7% of fathers were employed, 3.7% were out of work, and 0.6% were retired. A total of 84% of mothers did not work, 15.9% had a job, and 0.1% were retired. Most adolescents (89.9%) were living with both parents, 8.7% with a single parent, and 1.4% were living alone or with other relatives.

### Measures

#### The Inventory of Parent and Peer Attachment (IPPA-45)

The IPPA-45 [79] is a 45-item short form of the IPPA [6], comprising three 15-item scales that assess maternal, paternal, and peer attachment quality. Each scale has three subscales: trust, communication, and alienation. A sample item from the peer scale reads “*My friends respect my feelings*,” and respondents were asked to rate each item for how true it is for them on a Likert scale ranging from 1 (*Almost never or never*) to 5 (*Always or almost always*).

#### The behavior problems Youth Self-Report (YSR)

The YSR [1] is a self-report measure of behavior problems among adolescents. The internalizing comprises three subscales: a) anxious/depressed, b) withdrawal/depressed, and c) somatic complaints. As part of the externalizing scale, two aspects of behavior are assessed: a) breaking the rules, and b) being aggressive. Respondents are asked to rate how true each item is of them using a Likert scale from 0 (*not true*) to 2 (*very true or often true*). The present study used a standardized Persian version of YSR [20]. Cronbach’s alpha values of 0.92 and 0.91 were determined for internalizing and externalizing problems, respectively, in the current study.

#### Academic performance

Academic performance was assessed using a self-report measure that asked participants to rate their perceptions of their school performance. The single item asked: “*How do you evaluate your academic performance*?” using a scale from 1 (*poor performance*) to 5 (*excellent performance*).

#### Demographic characteristics

An ad hoc questionnaire was developed to gather information on demographic characteristics of participants, including gender (male/female), fathers’ and mothers’ educational level (illiterate/primary school/secondary school/diploma/associate degree/bachelor’s degree/master’s degree/PhD degree), fathers’ and mothers’ occupation (employee/freelancer/worker/unemployed/retired), the adult(s) they live with (both parents/mostly with mother/mostly with father/with mother and father alike/with adults who are not their parents/alone), and age.

### Procedure

Translation of the IPPA-45 scale into the Persian language was conducted by a bilingual team of professional translators comprising three mental health experts and a linguist, followed by back-translation into English [33]. After reviewing the back translation, an English language expert confirmed that the translation remained consistent with the original. An initial pilot study was conducted to assess the comprehensibility of the translated scale. Thirty high school student volunteers (15 girls) were asked to rate the clarity of the items on a scale from 0 (*not understandable*) to 5 (*completely understandable*). Approximately 97% of adolescents found the items comprehensible. Note that these students did not participate in the main study.

Data collection was carried out following approval from the Ethics Committee of the Iran University of Medical Sciences (Approval ID: IR.IUMS.REC.1400.190). Invitations to participate in the study were extended to 16 schools, and six accepted. A statement explaining the study’s purpose was presented to students, informing them that participation was voluntary. Consent forms were distributed to both students and their parents once they volunteered to participate. Out of a total of 1,800 students enrolled in the six schools, 1535 (86.27%) students and their parents agreed to participate in the survey. Participants were verbally informed of their right to withdraw from the research at any time and were asked to answer the questions honestly. To complete the IPPA-45 and YSR scales, students were provided with an online link. Ultimately, 1532 students completed all the scales.

### Statistical analysis

Data screening was conducted using IBM SPSS Statistics Version 28 (SPSS citation IBM Corp, 2021). Homogeneity of variance was assessed for all the IPPA-45 items and confirmed using Levene’s test. All items met the univariate outlier criteria (|z|≤ 2.00), and the original mean was compared with a 5% trimmed mean to evaluate the impact of potential outliers. Outliers were retained in the analysis, as they did not substantially affect the central tendency. Robust estimation methods, as recommended by Tabachnick et al. [68], were employed to ensure the reliability of statistical parameters. Skewness and kurtosis values for all variables were calculated and fell within acceptable limits (e.g., ± 2), indicating approximate normality.

Next, Mplus 8.1 [55] was applied to conduct CFA with the robust maximum likelihood (MLR) estimator, to reduce bias and improve accuracy when testing prior factor structure models. Using Mahalanobis Distance (*p* < 0.001) [26], three out of 1535 cases were excluded due to multivariate normality [31]. As a part of our evaluation of the models’ “*goodness-of-fit*”, fit indices and their acceptable values were examined: the comparative fit index (CFI > 0.95), the Tucker-Lewis index (TLI > 0.95), root mean square error of approximation (RMSEA < 0.06) and its 90% confidence interval (CI), normal chi-square (*χ*^*2*^/df < 5), and the standardized root mean square residual (SRMR < 0.05) [39, 41, 49]. To further compare the competing models’ fit, the sample-size adjusted Bayesian information criterion (SSA-BIC) was applied, in which a lower value represents a more accurate model.

Second, the selected factor structure model underwent a measurement invariance test across gender to evaluate the equivalence of the configural (same pattern of factor loadings), metric (equal factor loadings), and scalar (equal intercepts) models. To do so, the similarity of RMSEA values and their CIs, as well as discrepancies in CFI, RMSEA, and SRMR for nested models, was examined. Two conditions in fit indices were required to establish invariance: ∆CFI ≤ 0.01, ∆SRMR ≤ 0.03, and ∆RMSEA ≤ 0.015 for metric invariance and ∆CFI ≤ 0.01, ∆SRMR ≤ 0.01, and ∆RMSEA ≤ 0.015 for scalar invariance [14, 63]. Third, reliability was evaluated using R version 4.3.0 [69] by means of inter-item correlation, Cronbach’salpha, and its equivalents for Likert-type ordinal measures, such as ordinal alpha, omega, ordinal theta, and composite reliability [7, 13, 83]. According to Cicchetti [15], a correlation of 0.70 or higher demonstrates a satisfactory level of internal consistency. The modified item-total correlation was used to indicate items’ discrimination (0.01–0.19 = *not discriminating well*, 0.2–0.39 = *good discrimination*, and > 0.4 = *excellent discrimination*) [22].

Fourth, the internal construct validity of the IPPA-45 was assessed through the intercorrelations of subscales. Fifth, the convergent validity of the IPPA-45 was examined through its Spearman correlation (due to non-normality in data) with internalizing and externalizing behavior problems, academic performance, and age. Following the criteria proposed by Cohen [17], the correlations’ effect sizes were considered as 0.10 = small, 0.30 = medium, 0.50 = large, and 0.70 = very large. Furthermore, discriminant validity was examined using the Average Variance Extracted (AVE), which compares the variance in items captured by a latent variable with the variance due to measurement error [38]. AVE ≥ 0.5 indicates satisfactory discriminant validity [23]. Lastly, to examine group differences across gender while accounting for potential covariates, a Multivariate Analysis of Covariance (MANCOVA) was conducted. This analysis adjusted for the effects of age to ensure that observed differences in the IPPA-45 subscales were not confounded by age variability. Gender differences were evaluated for each subscale within the maternal, paternal, and peer forms. Effect sizes, reported as partial eta squared (*η*2), were included for all significant results. Effect size thresholds were defined as small (*η*^*2*^ = 0.01), medium (*η*^*2*^ = 0.06), and large (*η*^*2*^ = 0.14) [60].

## Results

### Factor structure and model selection

CFAs were conducted using Mplus version 8.9 (Muthén & Muthén, 1998–2023). In unidimensional models of 1–3 (M_1–3_), all items were assumed to load on a general factor of attachment for each maternal, paternal, and peer form [79]. Models 4–6 (M_4–6_) examined a three-factor oblique solution for each form.

**Fig. 1:**
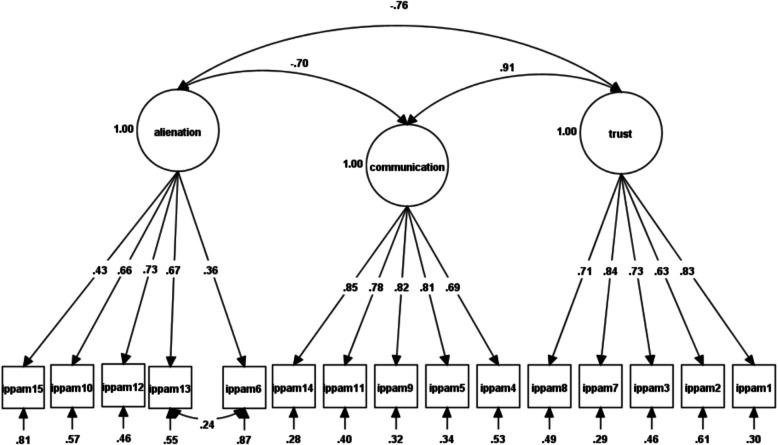
Factor loadings and latent factor covariances for Maternal first-order three-factor CFA after modification

**Fig. 2:**
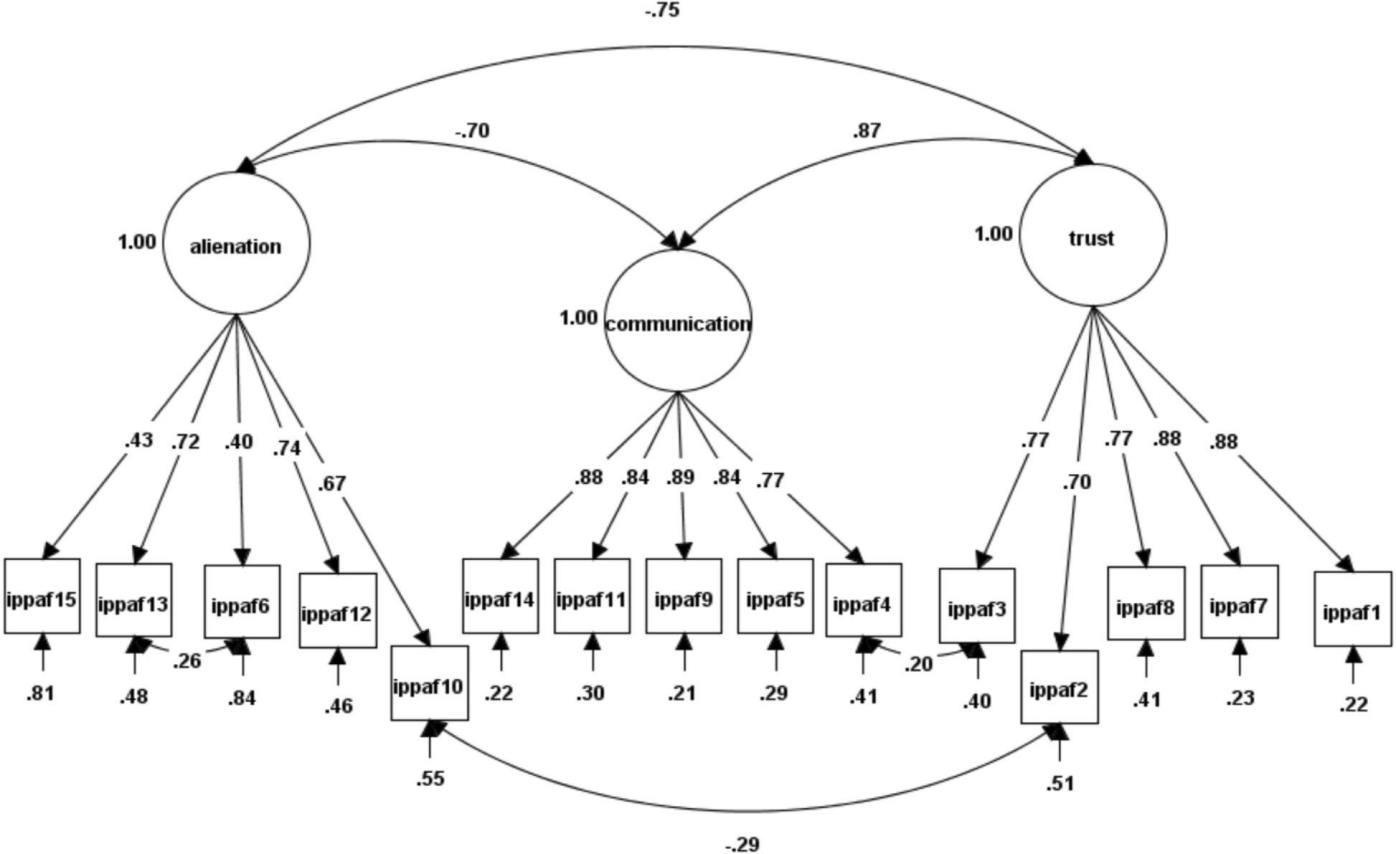
Factor loadings and latent factor covariances for Paternal first-order three-factor CFA after modification

**Fig. 3:**
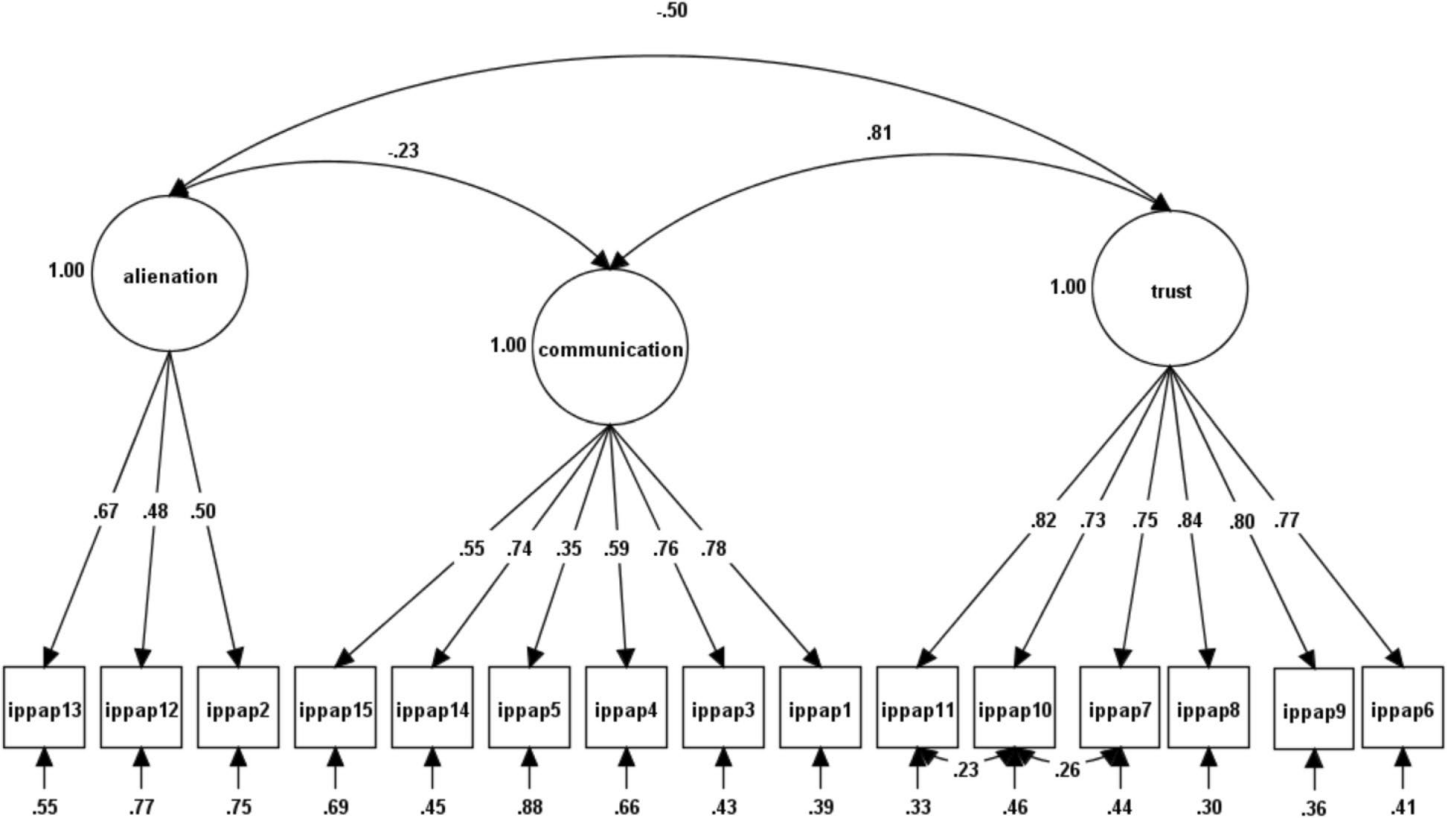
Factor loadings and latent factor covariances for Peer first-order three-factor CFA after modification

Table 1 indicates the fit indices of the one-factor models (M_1–3_). The hypothesized, oblique models for maternal (M_4_), paternal (M_5_), and peer forms (M_6_) were more acceptable. Still, with the CFI and TLI > 0.95 and RMSEA below 0.50, modifications were investigated based on modification indices (see Table 1 for more details). For the maternal form, modifications resulted in a better fit and represented a significant improvement compared to the hypothesized/original model [Fig. 1 and Table 1: modified M_4_; Δ*χ*^*2*^ = 59.22, df = 1, *p* < 0.001]. The father form was adjusted based on modification indices, and the modified model (modified M_5_ in Table 1) showed a significant improvement in fit [Fig. 2 and Table 1: modified M5; Δ*χ*^2^(180.44, df = 3, *p* < 0.001)], indicating an overall enhancement in goodness-of-fit. The hypothesized model for the peer form first did not fit well. Modification indices revealed a significant improvement in the model [Fig. 3 and Table 1: modified M6; Δ*χ*^2^ = 303.70, df = 2, *p* < 0.001], accompanied by enhanced goodness-of-fit.

**Table 1:** Confirmatory factor analysis of the inventory of parent and peer attachment (IPPA-45)

	Model	*χ*^*2*^	df	*χ*^*2*^/df	Scaling correction factor	CFI	TLI	SSA-BIC	RMSEA	SRMR	Base model	*Δχ*^*2*^(df)
One-factor Model	Maternal scale (M_1_)	1043.58^**^	91	11.46	1.30	.89	.87	62486.26	.08 (.07-.08)	.06	-	-
	Paternal scale (M_2_)	1678.92^**^	91	18.44	1.30	.85	.83	64901.21	.10 (.10-.11)	.07	-	-
	Peers scale (M_3_)	1188.08^**^	91	13.05	1.33	.85	.83	64459.50	.08 (.08-.09)	.09	-	-
Three-factor oblique Model	Maternal scale (M_4_)	485.84^**^	87	5.58	1. 28	.95	.94	61754.63	.05 (.05-.05)	.03	M_1_	558.01 (4)^***^
	Modified M_4_	430.99^**^	86	5.01	1.28	.96	.95	61689.07	.05 (.04-.05)	.03	M_4_	59.22 (1)^***^
	Paternal scale (M_5_)	676.86^**^	87	7.78	1.26	.94	.93	63615.53	.06 (.06-.07)	.04	M_2_	1002.06(4)^***^
	Modified M_5_	496.42^**^	84	5.90	1.25	.96	.95	63392.11	.05 (.05-.06)	.03	M_5_	180.44 (3)^***^
	Peers scale (M_6_)	1000.80^**^	87	11.50	1.29	.88	.85	64149.09	.08 (.07-.08)	.06	M_3_	187.28 (4)^***^
	Modified M_6_	404.50^**^	85	4.76	1.28	.96	.95	63385.36	.05 (.04-.05)	.04	M_6_	303.70.43(2)^***^

*χ*^*2*^ Satorra-Bentler adjusted *χ*^*2*^, *df* degrees of freedom, *TLI* Tucker–Lewis index, *CFI* comparative fit index, *SSA-BIC* sample-size adjusted Bayesian information criterion, *RMSEA* root mean square error of approximation, *SRMR* standardized root mean square residual, *χ*^*2*^/df normal chi-square, Δ*χ*^*2*^ difference between minus twice log likelihoods between the full and the nested models, Δdf difference in degrees of freedom between the full and nested models, Modified M_4_ model with correlated errors (items 6–13), *Modified* M_5_ model with correlated errors (items 6–13, 2–10 and 3–4), *Modified* M_6_ according to the modification indices, model with correlated errors (items 10–7 and 10–11) and items 4 and 5 were found to load on communication instead of alienation, while item 9 was reassigned to trust instead of communication

^**^ *p* <.01

^***^ *p* <.001

Overall, the IPPA-45 demonstrated satisfactory factorial validity, with minor adjustments to the parental forms and some more significant changes to the peer form. It generated three-factor first-order oblique structures for the maternal, paternal, and peer forms, which were generally consistent with the original full-item versions of the IPPA scales developed by Armsden and Greenberg [6] and the short-form versions proposed by Wilkinson and Goh [79]. Notably, the trust and communication scales exhibited higher loadings and better goodness-of-fit than the alienation scale.

### Measurement invariance across gender

First, to ensure a satisfactory fit for the baseline model in both boys and girls separately, a CFA was conducted for the entire sample as well as for each of the groups separately, according to the parsimony and meaningfulness perspective [76]. As shown in Table 2, a three-factor first-order oblique model was established as a baseline model and was run separately for boys and girls [76]. Afterwards, multi-group CFA analyses were conducted to evaluate configural, metric, and scalar invariances [10, 14].

**Table 2:** Invariance test across gender for the three-factor oblique model of the inventory of parent and peer attachment (IPPA-45)

Model invariance	*χ*^*2*^	df	*χ*^*2*^/df	scaling	CFI	TLI	SSA-BIC	RMSEA	SRMR	Base model	Δ*χ*^*2*^(df)	ΔCFI	ΔTLI	ΔRMSEA	ΔSRMR
Maternal Form															
Configural	539.67^**^	172	3.13	1.26	.96	.95	61689.06	.05 (.04-.05)	.03	-	-	-	-	-	-
Metric	553.15^**^	184	3.00	1.30	.96	.95	61656.80	.05 (.04-.05)	.04	Configural	13.73 (12)	.000	.004	.002	.008
Scalar	603.76^**^	196	3.08	1.27	.96	.95	61656.80	.05 (.04-.05)	.04	Metric	54.18 (12)^***^	.000	.000	.000	.000
Paternal Form															
Configural	576.24^**^	168	3.43	1.24	.96	.95	63385.19	.05 (.05-.06)	.03	-	-	-	-	-	-
Metric	590.76^**^	180	3.28	1.28	.96	.96	63347.20	.05 (.05-.06)	.04	Configural	10.91 (12)	.001	.003	.001	.002
Scalar	639.78^**^	192	3.33	1.22	.96	.95	63348.39	.05 (.05-.06)	.04	Metric	51.20 (12)^***^	.003	.001	.000	.002
Peers Form															
Configural	489.83 ^**^	170	2.88	1.27	.96	.95	63402.52	.05 (.04-.05)	.04	-	-	-	-	-	-
Metric	516.10^**^	182	2.83	1.27	.96	.95	63382.42	.04 (.04-.05)	.05	Configural	25.52 (12)^*^	.002	.001	.001	.006
Scalar	592.42^**^	194	3.05	1.25	.95	.94	63420.28	.05 (.04-.06)	.05	Metric	86.27 (12)^***^	.009	.006	.003	.009

*χ*^*2*^ Satorra-Bentler adjusted *χ*^*2*^, *df* degrees of freedom, *TLI* Tucker–Lewis index, *CFI* comparative fit index, *SSA-BIC* sample-size adjusted Bayesian information criterion, *RMSEA* root mean square error of approximation, *SRMR* standardized root mean square residual, *χ*^*2*^/df normal chi-square, Δ*χ*^*2*^ difference between minus twice log likelihoods between the full and the nested models, Δdf difference in degrees of freedom between the full and nested models

^*^ *p* <.05, ^**^ *p* <.01, ^***^ *p* <.001

For the three-factor oblique model, configural invariance was established across gender, as only minimal variations in the model fit indices were observed. According to Table 2, the hypothesized invariances of the IPPA-45 (i.e., three-factor first-order oblique model) fitted the data well. This reveals that the factor structure remains consistent (equal forms) across gender for maternal (*χ*^2^ = 539.68, CFI = 0.96, RMSEA = 0.053), paternal (*χ*^2^ = 576.25, CFI = 0.96, RMSEA = 0.056), and peers’ forms (*χ*^2^ = 489.83, CFI = 0.96, RMSEA = 0.050). This comparable factor loadings across gender for maternal (Δ*χ*^2^(df) = 13.73(12), *p* > 0.05) and paternal (Δ*χ*^2^(df) = 10.91(12), *p* > 0.05) forms, but not for peers’ form (Δ*χ*^2^(df) = 25.52(12), *p* < 0.05). The results indicated non-equivalent item intercepts across gender for maternal (Δ*χ*^2^(df) = 54.18(12), *p* < 0.001), paternal (Δ*χ*^2^(df) = 51.20(12), *p* < 0.001), and peer (Δ*χ*^2^(df) = 86.27(12), *p* < 0.001) forms.

### Internal reliability

In Table 3, descriptive statistics, Cronbach’s alpha, ordinal alpha, omega, ordinal theta, and composite reliability coefficients, as well as corrected item-total correlations, are presented for each of the IPPA-45 subscales. Almost all subscale items exhibited moderate corrected item-total correlations, ranging from 0.32 to 0.85. Lastly, the average inter-item correlations for the subscales of trust, communication, and alienation in the father’s, mothers, and peers’ forms ranged from 0.34 to 0.71, except for alienation with peers (0.30; Table 2).

**Table 3:** Items’ and scales’ level descriptive statistics, and reliability of the inventory of Parent and Peer Attachment (IPPA-45)

Form	Subscale	*r*^cs^	MIIC	Reliability					AVE	Mean and SD		
				Alpha	Ordinal alpha	Ordinal theta	Omega	Composite		Total M (SD)	Male M (SD)	Female M (SD)
Maternal	Trust	.54-.77	.49	.87	.91	.88	.88	.91	.60	28.73 (5.73)	29.35 (5.16)	28.11 (38.29)
	Communication	.64-.79	.62	.89	.92	.90	.90	.92	.70	18.84 (5.29)	19.23 (4.93)	18.46 (5.60)
	Alienation	.36-.50	.34	.61	.66	.68	.68	.69	.46	8.13 (2.97)	8.26 (2.93)	8.006 (3.02)
Paternal	Trust	.54-.82	.58	.90	.93	.91	.91	.93	.69	23.39 (5.92)	23.84 (5.53)	22.94 (6.27)
	Communication	.72-.85	.71	.92	.95	.93	.93	.95	.78	16 (6.09)	17.08 (5.74)	14.90 (6.23)
	Alienation	.42-.61	.37	.70	.76	.72	.72	.73	.46	10.44 (4.01)	10.38 (3.92)	10.50 (4.10)
Peer	Trust	.70-.80	.63	.91	.93	.92	.92	.93	.74	23.15 (5.55)	23.03 (5.23)	23.27 (5.85)
	Communication	.32-.67	.40	.79	.82	.82	.82	.83	.53	19.69 (5.34)	19.30 (5.15)	20.07 (5.50)
	Alienation	.34-.42	.30	.57	.64	.55	.58	.63	.31	7.00 (2.58)	6.97 (2.50)	7.00 (2.65)

*M* mean, *SD* standard deviation, *r*^cs^ corrected item-total correlation for subscales’ items, *MIIC* mean of inter-item correlations, *Alpha* standard Cronbach’s alpha, *AVE* average variance extracted

### IPPA-45 subscale inter-correlations

The internal construct validity was supported through the intercorrelations among the subscales of the IPPA-45 (Table 4). All trust and communication subscales showed significant positive correlations with each other, with coefficients ranging from 0.09 (between maternal trust and peer communication) to 0.80 (between maternal trust and communication, and between paternal trust and communication). The alienation subscale in all three forms also had significant and positive correlations with each other (*r* = 0.25 to 0.43,* p* < 0.01). Alienation had negative correlations with trust and communication subscales in three forms, with the strongest correlation between alienation and communication in the paternal form (*r* = −0.47,* p* < 0.01) and the weakest non-significant association between maternal alienation and peer communication (*r* = −0.003, *p* > 0.01).

**Table 4:** Correlation matrix of the inventory of parent and peer attachment (IPPA-45), gender, academic performance, and behavior problems (*N* = 1531)

	1	2	3	4	5	6	7	8	9
1. Maternal trust	1								
2. Maternal communication	.80^**^	1							
3. Maternal alienation	-.04	-.13^**^	1						
4. Paternal trust	.58^**^	.52^**^	-.16^**^	1					
5. Paternal communication	.55^**^	.60^**^	-.23^**^	.80^**^	1				
6. Paternal alienation	-.35^**^	-.36^**^	.43^**^	-.41^**^	-.47^**^	1			
7. Peer trust	.29^**^	.24^**^	-.09^**^	.30^**^	.28^**^	-.15^**^	1		
8. Peer communication	.09^**^	.09^**^	.003	.10^**^	.10^**^	-.02	.70^**^	1	
9. Peer alienation	-.24^**^	-.19^**^	.25^**^	-.21^**^	-.19^**^	.40^**^	-.36^**^	-.20^**^	1
10. Anxious/Depressed	-.42^**^	-.37^**^	.24^**^	-.39^**^	-.41^**^	.40^**^	-.24^**^	-.07^**^	.37^**^
11. Withdrawal/Depressed	-.46^**^	-.47^**^	.25^**^	-.42^**^	-.50^**^	.41^**^	-.23^**^	-.12^**^	.31^**^
13. Somatic complaints	-.43^**^	-.38^**^	.20^**^	-.43^**^	-.44^**^	.34^**^	-.19^**^	-.04	.30^**^
14. Internalizing problems	-.49^**^	-.45^**^	.26^**^	-.46^**^	-.50^**^	.43^**^	-.25^**^	-.09^**^	.37^**^
15. Rule-breaking behavior	-.37^**^	-.35^**^	.12^**^	-.33^**^	-.30^**^	.25^**^	-.16^**^	-.03	.21^**^
16. Aggressive behavior	-.47^**^	-.43^**^	.17^**^	-.43^**^	-.42^**^	.34^**^	-.22^**^	-.04	.33^**^
17. Externalizing problems	-.46^**^	-.42^**^	.16^**^	-.42^**^	-.40^**^	.33^**^	-.21^**^	-.04	.30^**^
18. Behavior problems total	-.52^**^	-.48^**^	.24^**^	-.50^**^	.43^**^	.49^**^	-.26^**^	-.07^**^	.37^**^
19. Age	-.06^*^	-.09^**^	.09^**^	-.09^**^	-.13^**^	.10^**^	-.08^**^	-.07^**^	.11^**^
20. Academic Performance	.30^**^	.28^**^	-.14^**^	.32^**^	.32^**^	-.26^**^	.19^**^	.11^**^	-.20^**^

^*^ *p* <.05, ^**^ *p* <.01, ^***^ *p* <.001

### Convergent and discriminant validity of IPPA-45

Convergent validity was evaluated by examining the relationship between the IPPA-45 and its subscales, as well as internalizing and externalizing behavior problems (Table 4). The Spearman correlation coefficients showed that internalizing problems (anxious/depressed, withdrawal/depressed, and somatic complaints) had significant and negative relationships with trust and communication in parents (*r* = −0.37 to −0.50, *p* > 0.01), and peer forms (*r* = −0.07 to −0.25, *p* > 0.01), while they showed significant and positive relationships with alienation in parents (*r* = 0.20 to 0.43, *p* > 0.01), and peer forms (*r* = 0.30 to 0.37, *p* > 0.01). Externalizing problems (rule-breaking behavior and aggressive behavior) manifested significant and negative relationships with trust and communication in parents (*r* = −0.30 to −0.46, *p* > 0.01), and peer forms (*r* = −0.16 to −0.21, *p* > 0.01), as well as significant and positive relationships with alienation in parents (*r* = 0.12 to 0.34, *p* > 0.01), and peer forms (*r* = 0.21 to 0.33, *p* > 0.01).

The teenagers’ age was negatively correlated with subscales of trust and communication in maternal, paternal, and peer forms (*r* = −0.06 [*p* < 0.01] to −0.13 [*p* < 0.05]), while it had a positive correlation with maternal, paternal, and peer alienation (*r* = −0.09 to 0.11, *p* < 0.01). Educational performance, on the other hand, had positive correlations with the subscales of trust and communication in maternal, paternal, and peer forms (*r* = 0.11 to 0.32, *p* < 0.01), but a negative correlation with maternal, paternal, and peer alienation (*r* = −0.14 to −0.26, *p* < 0.01).

Furthermore, the trust and communication subscales in three forms of parent and peer attachment had an *AVE* of ≥ 0.5, indicating acceptable discriminant validity. However, the subscale of alienation in all forms had *AVE* < 0.5, questioning the discriminant validity of these subscales.

### Gender differences in parent and peer attachment

Table 3 presents the mean scores and standard deviations of the IPPA-45 subscale across gender. A Multivariate Analysis of Covariance (MANCOVA) of maternal form revealed significant group differences by gender [F(3, 1526) = 12.41, *p* < 0.001, η2 = 0.03] in mean scores of the subscales, after adjusting for the age effect. Subsequent tests of between-subjects effects showed that boys scored significantly higher than girls on trust [*F* (1–1530) = 20.06, *p* < 0.001, *η*^2^ = 0.01], communication [*F* (1–1530) = 8.29, *p* = 0.004, *η*^2^ = 0.005], and alienation [*F* (1–1530) = 15.12, *p* = 0.001, *η*^2^ = 0.01]. The analysis of paternal form revealed significant group differences by gender [*F* (3, 1526) = 26.75, *p* < 0.001, *η*2 = 0.01] in mean scores of the subscales, after adjusting for the age effect. Subsequent tests of between-subjects effects of paternal form showed that boys scored significantly higher than girls on trust [*F* (1–1530) = 9.44, *p* = 0.003, *η*^2^ = 0.01] and communication [*F* (1–1530) = 50.62, *p* < 0.001, *η*^2^ = 0.032]. The analysis of peers’ forms revealed significant group differences by gender [F (3, 1526) = 3.50, *p* < 0.014, *η*2 = 0.01] in mean scores of the subscales, after adjusting for the age effect. Subsequent tests of between-subjects effects for peers’ form showed that girls scored significantly higher than boys on communication [*F* (1–1530) = 8.25, *p* = 0.005, *η*^2^ = 0.01].

## Discussion

The primary objective of the current study was to assess the psychometric soundness of the Persian version of the IPPA-45 and to investigate the relationship between attachment to important figures in adolescence (parents and peers) and the internalizing and externalizing behavior problems of Iranian youth. The results revealed that the original three-factor model captured the best-fitting solution for the father, mother, and peer forms. Each form also demonstrated measurement equivalence across gender. Additionally, high internal reliability values were obtained from a comprehensive set of reliability tests across all three forms, establishing the strong internal consistency of the inventory. Findings also provided evidence for the convergent validity of the IPPA-45 by revealing a moderate negative relationship between adolescents’ attachment and internalizing and externalizing behavioral problems.

To our knowledge, no published validation study has evaluated the construct validity of the IPPA-45 in an adolescent sample to date. CFA results demonstrated that, in terms of the factorial structure, the modified original model with three correlated dimensions offered the best fit. The first-order model (consisting of trust, communication, and alienation) of the three forms of inventory (father, mother, and peer) was supported in the present study, allowing the use of subscale scores to measure paternal, maternal, and peer attachment security. Our findings are consistent with the factor structure proposed in the original research by Wilkinson and Goh [79].

The study yielded evidence showing that the IPPA-45 provided invariant measurement across gender (equal factor structure and factor loadings), implying that the same construct is measured among males and females. Accordingly, the number of factors and the pattern of factor indicators were similar across genders. The metric of factor scores was demonstrated to be identical, allowing unbiased comparison of factor means. Additionally, all components of the IPPA-45 were perceived similarly by male and female respondents, with no differential interpretation of its subscales (trust, communication, and alienation) across gender. Our findings are consistent with the research of Wilkinson and Goh [79], which proposes an invariant final model across genders.

Multiple reliability coefficients were reported for the internal consistency of the inventory, with each test having its own strengths and considerations. Cronbach’s alpha accounts for linear correlations among items, while omega assumes the presence of non-linear relationships. Ordinal theta and ordinal alpha are suitable for ordinal data, whereas composite reliability synthesizes measurement error and factor loadings [8, 80]. Cronbach’s alpha, omega, ordinal theta, ordinal alpha, composite reliability, and corrected item-total correlation estimates for the subscales of trust, communication, and alienation indicated acceptable to high levels of internal reliability. These results were in line with the findings of Wilkinson and Goh [79] in developing and evaluating Cronbach’s alpha of the IPPA-45. It is worth nothing that peer alienation had Cronbach’s alpha, omega, and ordinal theta < 0.60, which might be due to a lower item-total correlation of item five (i.e., “*My friends don’t understand what I’m going through these days*”*)* in peer form, compared to other items in the inventory.

The present study provided evidence of the internal construct validity of the IPPA-45 in its mother, father, and peer versions. Regarding subscales intercorrelations in all three forms of the IPPA-45, a positive correlation was found among all dimensions, with the exception of the negative correlation between the alienation subscales and all other dimensions. These findings are consistent with Wilkinson and Goh’s [79] results, which demonstrated high internal consistency and acceptable to high intercorrelations between the subscales in all three forms. It is also worth noting that, in line with a longitudinal quantitative study conducted by Ridenour et al. [61], parental alienation gradually results in a lack of trust and weakened parent–child communication, which justifies the existing negative association within these subscales. Moreover, the strong association between trust, communication quality, and alienation subscales could be explained by the idea that difficulties in one aspect of the parent-teen relationship often coincide with challenges in other areas [16].

Another primary study goal involved testing the convergent validity of the IPPA-45 through its associations with other constructs that are considered logical outcomes of attachment. The results showed a significant negative correlation between parental and peer attachment and all subscales of behavioral problems. Although all correlations were weak to moderate, peer attachment domains correlated more weakly with behavioral problems compared to father and mother attachment security domains. Trust and communication, compared to alienation, were more strongly associated with behavioral problems in all three forms. Notably, the father’s alienation manifested the strongest correlation coefficient with both internalizing and externalizing problems when compared with the mother’s and peers’ alienation, which highlights its underlying role in subsequent youth behavioral dysfunction. Previous research has revealed the existing relationship between attachment dimensions and behavioral problems such as internalizing/externalizing problems [2], aggressive behavior [64], and withdrawal/depressed subscale [42]. In line with attachment theory [9], dysfunctional rage or aggressiveness is at the core of insecure attachment, indicating that individuals with insecure attachment suffer from a contradiction between their fundamental desire for closeness and their expectations regarding the responsiveness of others. These results align with the existing international literature on attachment and various problematic behaviors, including research conducted by Guedes et al. [32], Vagos and Carvalhais [70], and Wilde [77].

Different aspects of attachment varied for adolescents’ age and academic performance in high school. Results showed a positive association between academic performance and positive attachment dimensions. Our findings also indicated that the adolescent’s age had a negative and significant relation with mother, father, and peer attachment security. Similarly, Nickerson and Nagle [56] found that younger adolescents had more trusting and communicative relationships with their parents than older adolescents, which is consistent with the negative relationship between trust and communication subscales and age found in the present study. Furthermore, the trust and communication subscales in three forms of parent and peer attachment had an AVE higher than 0.5, indicating an acceptable discriminant validity. However, the subscale of alienation in all forms had AVE < 0.5, which may mean that discriminant validity for these subscales is not established at the construct level, and their items explained more errors than the variance in the subscale [38].

While there was measurement equivalence between boys and girls, the overall gender-specific patterns in terms of the IPPA-45’s subscales were evident. According to the findings, boys scored significantly higher than girls on trust and communication in both maternal and paternal forms of the IPPA-45. The girls, however, scored significantly higher on the communication subscale than the boys for the peer form. Generally, parents respond differently to their children based on sex, and unconscious biases can impact how parents treat sons or daughters [51]. In traditional Eastern societies such as Iran, an authoritarian parenting style is prevalent, and existing cultural influences lead parents to adopt a less engaging and more authoritarian role in relationships with their children, especially with their daughters [59]. Generally, boys receive less strictness, leading them to interact more with their parents [29], which may explain why girls score lower on trust and communication than boys in the parental form. Girls may also seek compensatory routes and communicate more with peers. In the same vein, Wilkinson and Goh [79] found that boys have marginally higher scores on trust items than girls.

### Limitations and future directions

In the present study, we developed the Persian-language version of the IPPA-45 in a cross-cultural setting and demonstrated satisfactory psychometric properties. The Persian edition is likely to be used in various settings throughout Iran and other Persian-speaking countries. However, several significant limitations must be acknowledged. First, only adolescents aged 14–17 living in a central metropolitan area (Tehran) were included in the present study; thus, it is crucial to proceed with caution when applying these findings to different developmental stages or teenagers living in rural regions. Second, the data were collected via self-report inventories rather than the multi-informant method; therefore, mono-informant may affect the reported results instead of accurately reflecting the intended constructs, and adolescents might have answered in a socially desirable way [58]. Utilizing a multi-informant method to achieve a broader overview of the adolescent’s attachment might be beneficial and informative. Third, the low item-total correlation of item five in the peer attachment form, which was related to peer alienation, resulted in moderate reliability indices for this subscale. Fourth, due to the cross-sectional nature of the study and the limited data from a homogenous population, causal relationships between attachment and other study variables cannot be precisely inferred. Fifth, obviously, no single measurement can fully tap either the variety of attachment relationships in adolescence or the complexity of the fundamental shifts in attachment processes that are occurring. Thus, the impact of other plausible relationships, such as romantic partnerships and best friendships, as well as the effect on the quality of interactions with others in the social network, might be overlooked. Given the dramatic fluctuations associated with puberty, which can impede parents’ communication with their children, adolescents may experience alterations in their relationships with their parents, as well as changes in their perception of security and attachment figures over time [21]. Therefore, it is proposed that future research should include a longitudinal examination of adolescents’ perceptions of parental security. Lastly, another limitation of interpreting the correlation analysis is that the large sample size may result in statistically significant correlations even when effect sizes are small. As a result, while the correlations are significant, their practical significance should be interpreted with caution.

### Clinical and research implications

The current findings offer several implications for research and practice. The first implication is to consider gender differences in attachment patterns when providing community-based health services or clinical interventions to adolescents. Thus, healthcare practitioners are not misled by biological sex effects and would not expect the same outcomes for boys and girls. Secondly, given that the results revealed a significant correlation between externalizing/internalizing behavioral problems and dominant attachment patterns, specialists should be aware of the potential predisposing role of attachment patterns in the development of externalizing and/or internalizing behavioral problems when interacting with adolescents. As a result, being aware of the attachment pathway to behavioral problems highlights the significance of attachment-based interventions that aim to strengthen parental abilities to provide responsive and sensitive care, while also serving as a preventive measure for future behavioral difficulties. The third implication of our findings is that attachment patterns may not be consistent and stable across the lifespan. The strength of attachment relationships may fluctuate as indicated by the negative correlation between age and attachment security. Subsequently, healthcare practitioners and community-based specialists must be aware of these modifications and avoid neglecting the effects of various developmental stages and puberty when studying adolescents. Finally, it is noteworthy that the attachment security of teens toward their peers was linked with behavior problems. This suggests that when targeting attachment insecurity or behavior problems in adolescents, interventions should not be limited to mother and father attachment but also take into consideration their sense of being attached to their peer groups.

## Conclusion

Taken together, the results of this study provide support for the construct validity, gender equivalence, reliability, convergent validity, and discriminant validity of the Inventory of Parent and Peer Attachment-short form. Attachment relationships with parents and peers were negatively correlated with internalizing and externalizing behavior problems, as well as age, while they were positively associated with academic performance. Distinctive patterns were found across gender, with boys having stronger secure relationships with their parents and girls being more attached to their peers. Stricter parenting for daughters and more lenient, interactive parenting for sons lead to lower parental trust and communication for girls, who compensate with stronger peer connections. This study may also encourage researchers to further examine the psychometric properties and practicality of the IPPA-45 in clinical samples. Additional studies could investigate the replicability of the inventory and its factor structure in different cultural contexts. Furthermore, our results provided insight into designing interventions for adolescents with internalizing and externalizing behavior problems, particularly targeting attachment relationships, considering gender differences, and taking into account the importance of peer attachment.

## Data Availability

The raw data supporting the conclusions of this article can be provided by the corresponding author upon reasonable request.
